# Multifocal PEComa (PEComatosis) of the female genital tract and pelvis: a case report and review of the literature

**DOI:** 10.1186/1746-1596-7-23

**Published:** 2012-03-09

**Authors:** Wang Yang, Gao Li, Zheng Wei-qiang

**Affiliations:** 1 Department of Pathology, Changhai Hospital, Second Military Medical University, Shanghai 200433, China

**Keywords:** Perivascular epithelioid cell tumor (PEComa), PEComatosis, Immunohistochemistry

## Abstract

Perivascular epithelioid cells (PECs) are constantly present in a group of tumors called PEComas, including angiomyolipoma (AML), clear-cell "sugar" tumor (CCST) of the lung and extrapulmonary sites, lymphangioleiomyomatosis, clear-cell myomelanocytic tumor of the falciform ligament/ligamentum teres and rare clear-cell tumors of other anatomic sites. PECs have distinctive morphologic, immunohistochemical, ultrastructural and genetic characteristics, including an epithelioid appearance with a clear to granular cytoplasm, a round to oval, centrally located nucleus, and an inconspicuous nucleolus. PECs also express melanocytic and myogenic markers like HMB45 and smooth muscle actin. PEComa is rare in human, and multifocal PEComas (PEComatosis) is extremely rare. Up to now there have been only less than 5 cases described in the literature. Here we report a 46-year old Chinese woman who had PEComatosis arising from the genital tract and pelvis. To our knowledge, this is the first case of PEComatosis ever reported in China.

**Virtual Slides:**

The virtual slide(s) for this article can be found here: http://www.diagnosticpathology.diagnomx.eu/vs/1293097548652023.

## 

Perivascular epithelioid cell tumor (PEComa) refers to a family of neoplasms showing at least partial morphological or immunohistochemical evidences of a putative perivascular epithelioid cell (PEC) differentiation. PEComa may involve the visceral organs, including the lung, kidney, liver, nasal cavity, small and large bowel, appendix, prostate and uterus, and also the retroperitoneal and abdominopelvic sites. The currently reported PEComas include renal and extrarenal angiomyolipoma (AML), lymphangioleiomyomatosis (LAM), clear cell "sugar" tumor of the lung (CCST), clear cell myomelanocytic tumors (CCMMT) of the falciform ligament/ligamentum teres and distinctive clear cell tumors at other anatomic sites. PECs have a clear or acidophilic cytoplasm and express the biomarkers of melanocytic and smooth muscle. The mean age of patients with PEComa is reportedly 45 years (ranging 40-75 years old), and there is a marked female predominance [1]. Here we describe a 46-year-old Chinese woman who had multifocal perivascular epithelioid cell tumor (PEComatosis) arising from the genital tract and pelvis.

## Case report

In Nov, 2010, a 46-year-old woman was referred to Department of Ggyneco-obstetrics of Changhai Hospital due to a uterus mass and a left pelvic adnexal mass, which were palpated during a routine physical examination, but presenting no abnormal vaginal bleeding and stigmata of the tuberous sclerosus complex. Serum CA-125 and CA-19-9 levels as well as other laboratory findings were all within the normal limits. Transabdominal sonography revealed a uterus mass (diameter 3.2 cm), a left pelvic adnexal cystic mass (diameter 3.7 cm) and a left pelvic mass(diameter 4.9 cm). The initial clinical impression of the lesions was uterus leiomyoma and left ovary chocolate cyst. Then the patient underwent a total abdominal hysterectomy and bilateral salpingo-oophorectomy. Intraoperative findings included smooth surface masses arising from the posterior wall of the enlarged uterus (diameter 3 cm), right part of cervix (diameter 3 cm) and the left broad ligament (diameter 4 cm), with hypertrophic ovaries, normal fallopian tubes, without ascites. An intraoperative frozen section of the uterine mass was interpreted as leiomyoma.

**Figure 1:**
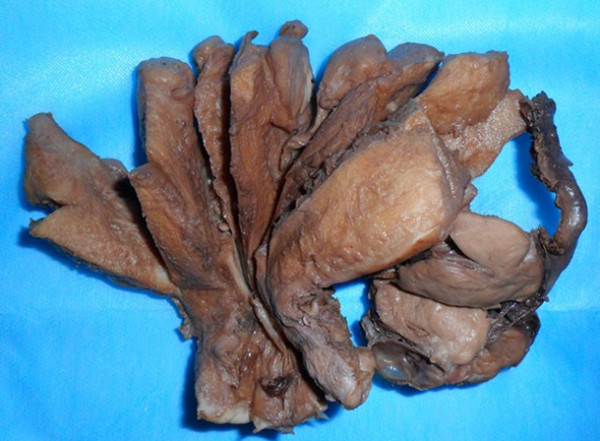
**The uterus had 2 myometrial nodules(arrows) and the left broad ligament had a lobulated nodule (star)**. No hemorrhage, necrosis or infiltrative features were seen in the uterine corpus, ovary, or fallopian tube.

**Figure 2:**
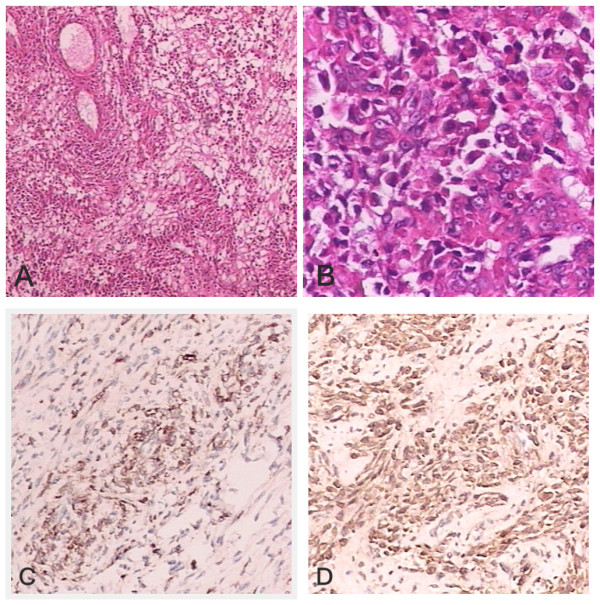
**(A) Epithelioid tumor cells are around the delicate vasculature (perivascular arrangement; H-E, ×40)**. (B) Perivascular epithelioid cells are round to polygonal with abundant clear to finely eosinophilic granular cytoplasm and round nuclei (H-E, ×200). The epithelioid cells are immunoreactive for HMB-45(C) and calponin(D), with the characteristic "myomelanocytic" immunophenotype of PEComas.

The surgical specimens were collected and fixed in 10% formalin for H-Estaining and immunohistochemical staining. The immunophenotypes of tumor cells was examined by EnVision Plus system (DAKO Corporation). The antibodies used included HMB-45 (1:50), Melan-A(1:50), smooth muscle actin (1:50), calponin (1:100), CD34 (1:50), CD10(1:100), S-100(1:200), p53(1:50), estrogen receptor (1:80), progesterone receptor (1:40) and MIB-1 (1:200). The surgical specimens included a 9 cm × 6 cm × 6 cm uterus with left adnexae and right cavitas pelvis masses. Grossly, two round masses were found in the uterine, with a 3 cm × 3 cm × 2 cm mass in the myometrium and a 3 cm × 3 cm × 3 cm mass in the subserosa of posterior wall; a mass measuring 5 cm × 5 cm × 4 cm was found in the left broad ligament, multiple masses were found in the right cavitas pelvis, with the largest diameter being 3 cm (Figure 1). The masses were round or nodulose, solid, well-demarcated and partially encapsulated by thin membranous tissue. The cross section surfaces of the masses were grayish-white, without fresh, old hemorrhage or necrosis. A 3 cm × 3 cm × 3 cm cystic tumor was found on the left adnexa. H-E staining showed that all the PEC lesions in our case had a similar morphological feature under microscope. The tumors were mainly composed of epithelioid cells, with a few spindle-shaped cells; the tumor cells had abundant clear-to-fine eosinophilic granular cytoplasm, with round to oval nuclei, mild-to-moderate pleomorphism and a mitotic count of 1-4/50HPF. There were no necroses; the tumor cells were arranged in nests, bundles or patches; some surrounded and radiated alone the blood vessels. And there were no pathological mitoses. Proliferation of thin-walled capillary-like vessels was also noted in foci with occasional glomerulus-like vascular tuft formation. There were no mature adipose tissues, spindle-shaped smooth-muscle bundles, or abnormal thick-walled blood vessels characteristic of classic AML and necrosis. Immunohistochemically, the tumor cells were negative for CD34, CD10, S-100 and p53, and strongly positive for HMB-45 (Figure 2C) and Melan-A, smooth muscle actin (SMA), calponin (Figure 2D), ER and PR. A low proliferative fraction was detected with MIB-1 antibody(3-5% positive MIB-1 staining). PAS staining without diastase predigestion found that some tumor cells were positive, and PAS staining with diastase predigestion showed completely negative results. Masson staining of the connective tissues showed a great deal of collagen fibers in the stroma.

Other specimens revealed chronic cervicitis, endometrium of proliferative phase, follicular cyst of left ovary, normal right ovary, and fallopian tubes. The postoperative course was uneventful, and the patient was discharged 4 days after surgery. No further treatment was needed; and no recurrence was noticed 12 months later as shown by transabdominal sonography of the abdomen and pelvis. She is currently followed up regularly by ultrasonography.

## Discussion

PECs, first described by Bonetti et al [2], do not have a normal anatomic homologue. PECs are characterized by co-expression of melanocytic and muscle markers, epithelioid to spindle shapes with ample clear to eosinophilic cytoplasm, and at least in some cases, arrangement around blood vessels [3]. The term "PEComa"(perivascular epithelioid cell tumor) was introduced in 1996 by Zamboni et al [4], as synonym for tumors composed primarily by PECs. In the WHO soft tissue volume, PEComas are defined as "mesenchymal tumors composed of histologically and immunohistochemically distinctive perivascular epithelioid cells" [5], including entities such as AML, LAM, and CCST of the lung. In recent years, more tumors are being reported and categorized as members of the PEComa family, including monotypic epithelioid AML, extrapulmonary CCST, and clear-cell myomelanocytic tumor (CCMMT) of the falciform ligament/ligamentum teres.

PEComas may arise from multiple anatomical sites, including the kidney, lung, pancreas, liver, uterus, breast, ligament teres, falciform ligament, orbit, skin and soft tissue, with the uterus and retroperitoneum appearing to be the most frequent sites of origin. The first case of uterus PEC tumor was reported by Pea et al [6]. PEComas share a common morphophenotypic feature: diffuse, nested and/or fascicular proliferation of spindled and epithelioid cells, tumor cells with clear to eosinophilic cytoplasm, centrally located nuclei, and immunoreactivities to both the melanogenic-related markers and to a lesser extent, muscular markers. Folpe et al [7] reported CD117 reactivity in about 5% of PEComas. Ultrastructurally, structures interpreted as melanosomes and premelanosomes have been demonstrated in PEComa of the uterus [8]. Vang and Kempson [9] divided uterine PEComas into A and B types. Type A tumors show a tongue-like growth pattern and are composed of cells with clear to eosinophilic pale granular cytoplasm, diffuse HMB-45 expression, and focal smooth muscle reactivity. Type B tumors are composed of epithelioid cells with less prominent clear cell features, with only a few of being HMB-45 positive; in addition, cells of type B tumor demonstrate an extensive muscle marker expression and a lesser degree of tongue-like growth pattern.

Uterine PEComa lacks characteristic clinical and imaging changes; the diagnosis mainly relies on pathological approach, and should be differentiated from the following tumors: (1) Epithelioid smooth muscle tumor(ESMT)- the ESMT cells are round, polygonal and spindle-shaped; the spindle-shaped cells had cigarette-like nuclei with rounded ends; there was typical smooth muscle transition; they are usually HMB45 negative, without characteristic capillarity network of bloodvessels. PEComa is supplied by rich blood vessels, and the tumor cells surrounded the blood vessels, forming into patches and nests; they are often HMB45 positive. It has also been reported that a small number of ESMT cells could be HMB45 positive, but Melan-A negative; therefore double positive for SMA and Melan-A indicate a higher probability of PEComa. (2) Endometrial stromal sarcoma(ESS)-the tumor cells are spindle-shaped with less cytoplasm and negative for HMB45; PEComa cells are large and round or polygonal in shape, with rich eosinophilc cytoplasm, and are HMB45 positive. Some PEComa patients also have lymphangioleiomyomatosis and tuberous sclerosis, which has not reported in ESS patients. (3) Uterine clear cell carcinoma (UCC)-the tumor is composed of cells with clear cytoplasm and a hobnail morphology, which forming into solid, ductal or papillary shapes, negative for HMB45 and positive for CK. (4) Metastatic renal clear cell carcinoma (MRCcc)-the cytoplasm is clear and polygonal shaped; the round tumor cells arranged into nest, alveolar, ductal, or papillary shapes, but with no perivessel structure; the tumor is HMB45 negative, and CK, EMA positive. (5) Paraganglioma-it should be differentiated with PEComa when the cytoplasm is clear. Paraganglioma cells are arranged in streaks, glands, or nests, with flat supporting cells lining around, and with distinct organoid and pseudorosette structure; besides, they are HMB45 negative, and NSE, Syn, CgA and NF positive; the supporting cells are positive for S-100 protein.

PEComas have been reported as predominantly benign. Greene [10] believed that uterine PEComas were tumors with uncertain malignant potentials. Folpe et al [7] reported PEComas in 26 patients with soft tissue and gynaecologic origin, and classified the tumors as "benign", "of uncertain malignant potential" and "malignant"; they also observed a significant association between tumor size > 5 cm, infiltrative growth pattern, high nuclear grade, necrosis and mitotic activity > 1/50 HPF with aggressive behavior of PEComas. The 2002 WHO soft tissue and bone book [5] states that PEComas have the following features: infiltrative growth, marked hypercellularity, nuclear enlargement, hyperchromasia, high mitotic activity, atypical mitotic figures, and coagulative necrosis, and therefore PEComas should be regarded as malignant. However, by now we have failed to understand the real behavior of these ubiquitous tumors, because some tumors with "benign" appearance have aggressive behavior and others with "malignant" appearance have indolent course. Late recurrences of the tumor have been reported, including one with lung metastasis 7 years after the primary tumor had been described [11].

Due to the multicentricity of the lesions, we favor the designation of PEComatosis proposed by Fadare et al [12]. PEComatosis is very rare. Table 1 summarizes the clinical properties of all the five PEComatosis cases reported in the English literature. All the 5 cases were women aged 39-70 years old (a mean of 54 years old). Most cases involved the uterus, cervix and the genital system, and were complicated with TSC. Our case was a 46 years old woman involving the uterus, cervix, broad ligament and pelvic cavity, but without evidence of TSC.

**Table 1:** Reported Cases of PEComatosis in the English Language Literature (5 Cases)

Reference	Age/Sex	Site/Maximum Size(cm)	Histopathology	Immunohistochemistry	Comments
Fadare 2004 [12]	41/F	Myometrium, small bowel lamina propria, ovarian hila/2.2	No cytological atypia, mitotic activity or necrosis.	HMB-45 positive	With TSC. No recurrence or metastases at 35 months' follow-up.
Salviato 2006 [13]	70/F	Diffuse peritoneum/6	Epithelioid and spindle cells, mild to moderate nuclear pleomorphism, focal areas of necrosis.	HMB45, melan-A, and SMA positive, desmin and S100 negative.	With a history of ancient hysterectomy for fibroids
Liang 2008 [14]	59/F	Pelvic lymph nodes, myometrium, cervix, and ovary/2.6	Marked nuclear pleomorphism, necrosis, 2 mitoses/50 HPF, occasional atypical mitosis, infiltrative borders.	HMB45, melan-A, SMA and ER positive.	With TSC.
Froio 2008 [15]	39/F	Uterine corpus, both ovaries, and the omentum/		HMB45, melan-A, SMA, ER and PR positive.	With TSC.
Lim 2011 [16]	59/F	Uterine, cervix, uterine corpus.	Mature adipose tissue, spindle cells, small nests of clear epithelioid cells		With TSC.

Whether the multiple foci of PEComas are truly multicentric or whether they originate from a single primary site remains to be further investigated. To the best of our knowledge, the present case is the first PEComatosis in China. Treatment of PEComas is challenging since chemotherapy and radiotherapy seem not effective, and surgery is currently the most important treatment of these tumors.

## Competing interests

The authors declare that they have no competing interests.

## Authors' contributions

WY first identified this case,retrieved clinical information and wrote the manuscript. GL and ZW-q proposed the studies and provided valuable insight during manuscript preparation. All authors read and approved the final manuscript.
